# Oxaliplatin-Induced Neuropathy: Genetic and Epigenetic Profile to Better Understand How to Ameliorate This Side Effect

**DOI:** 10.3389/fmolb.2021.643824

**Published:** 2021-05-07

**Authors:** Jacopo Junio Valerio Branca, Donatello Carrino, Massimo Gulisano, Carla Ghelardini, Lorenzo Di Cesare Mannelli, Alessandra Pacini

**Affiliations:** ^1^Histology and Anatomy Section, Department of Experimental and Clinical Medicine, University of Firenze, Firenze, Italy; ^2^Pharmacology and Toxicology Section, Department of Neuroscience, Psychology, Drug Research and Child Health (NEUROFARBA), University of Firenze, Firenze, Italy

**Keywords:** oxaliplatin, neuropathic pain, glial cells, genetic mechanisms, epigenetic mechanisms

## Abstract

In the most recent decades, oxaliplatin has been used as a chemotherapeutic agent for colorectal cancer and other malignancies as well. Oxaliplatin interferes with tumor growth predominantly exerting its action in DNA synthesis inhibition by the formation of DNA-platinum adducts that, in turn, leads to cancer cell death. On the other hand, unfortunately, this interaction leads to a plethora of systemic side effects, including those affecting the peripheral and central nervous system. Oxaliplatin therapy has been associated with acute and chronic neuropathic pain that induces physicians to reduce the dose of medication or discontinue treatment. Recently, the capability of oxaliplatin to alter the genetic and epigenetic profiles of the nervous cells has been documented, and the understanding of gene expression and transcriptional changes may help to find new putative treatments for neuropathy. The present article is aimed to review the effects of oxaliplatin on genetic and epigenetic mechanisms to better understand how to ameliorate neuropathic pain in order to enhance the anti-cancer potential and improve patients’ quality of life.

## Introduction

### The Anti-cancer Features of Oxaliplatin

In the last decades, despite early cancer diagnosis, cancer deaths rapidly increased (Kanavos, 2006). Among the most common cancer types, colorectal cancer is one of the most diffuse cancer in both genders worldwide. Indeed, it has been recently reported that more than 1.8 million new cases and about 0.9 million deaths were estimated in 2018, ranking the colorectal cancer third in terms of incidence and second in terms of mortality in both male and female (Bray et al., 2018). Therefore, the researchers are making great efforts in order to counteract this debilitating and mortal health concern.

The platinum-derived drugs are widely used as chemotherapeutic agents and, among these compounds, cisplatin was the first one patented (Rosenberg et al., 1969). However, the poor effects of cisplatin against different cancer types (Taylor and Filby, 2017), its side effects (Lokich, 2001), and the cancer resistance (Stordal and Davey, 2007) limited its use in chemotherapy leading physicians and researchers to explore new paths in order to improve the quality of life of patients.

Within this framework, new platinum analogs have been developed. Although other new platinum-derived drugs are under study (Johnstone et al., 2016; Dai and Wang, 2020), nowadays, carboplatin and oxaliplatin are the most widely used in clinical practice (Fischer and Ganellin, 2006).

Oxaliplatin, a third generation 1,2-diaminocyclohexane platinum compound patented in 1976, shows high efficacy against colorectal cancer (Wiseman et al., 1999). Recently, its efficacy has been found also in other cancer types including lung (Raez et al., 2010), gastric (Cunningham, 2006; Zhang et al., 2019), ovarian, and prostate cancer (Zhou et al., 2017).

The primary anti-cancer effect exerted by oxaliplatin therapy, as well as for the other platinum-derived drugs, is due to DNA damage. Indeed, it has been largely demonstrated that oxaliplatin forms intra-strand crosslinks with DNA, as the main mechanism for the induction of DNA lesions, inhibiting the proliferation of neoplastic cells (Culy et al., 2000). Furthermore, another intriguing anti-cancer effect of oxaliplatin was discovered by Tesniere and colleagues, demonstrating that oxaliplatin-treated CT26 colorectal cancer cells were able to release immunogenic signals that trigger an immune response, thus leading to an enhanced anti-cancer effect promoted by the immune system (Tesniere et al., 2010).

With respect to oxaliplatin pharmacokinetics, it has been reported that the platinum compound can interact with and bind to plasma proteins. In patients affected by cancer, the platinum-plasma protein binding increased around 70% after 2 h of infusion, growing up to 95% 5 days after infusion. However, it can be considered that the maximum platinum concentration is achieved during the first cycle of oxaliplatin administration and no accumulation was reported after single or multiple doses, except for erythrocytes where an accumulation has been found (Culy et al., 2000; Lévi et al., 2000). On the other hand, the main excretion of platinum products occurs mainly by the renal route, consisting in about 50% after 72 h, whereas the excretion by defecation is very low, ranging about 2% after 5 days (Culy et al., 2000).

However, even if the antineoplastic efficacy of oxaliplatin is overt, it also unfortunately generates a series of undesired side effects.

### The Side Effects of Oxaliplatin Administration

Among the DNA-interfering chemotherapeutic agents, platinum-derived drugs can potentially interact with normal cells with high proliferating turnover, thus altering their physiological features and leading to adverse side effects (Oun et al., 2018).

Over the years, many researchers have highlighted the deleterious events in different organs and tissues resulting from oxaliplatin treatment. For instance, it has been shown that oxaliplatin exhibits irritant properties that may lead to the occurrence of vesicant lesions (Foo et al., 2003; Kennedy et al., 2003; Kretzschmar et al., 2003).

In contrast to cisplatin, oxaliplatin is not nephrotoxic or ototoxic, but it produces various adverse effects, the main one is neurotoxicity. Oxaliplatin-dependent neurotoxicity can be acute and/or chronic, but both types give rise to neuropathic pain (Sałat, 2020). The acute type elicits a transient neuropathy that occurs in 90% of patients within a few hours of chemotherapy administration, lasts for a few days, and recurs with subsequent administrations. The main signs and symptoms of acute neuropathy are exacerbated by cold and consist of dysesthesia and paresthesia of the hands and feet. Motor symptoms may also occur, such as tetanic spasms, fasciculations, and prolonged muscular contractions. It has been demonstrated that acute neuropathy is caused by a Na_v_ channel activation that transiently induces nerve hyperexcitability (Gebremedhn et al., 2018). In 70% of cases, prolonged exposure to oxaliplatin induces a severe chronic peripheral neuropathy, with symptoms very similar to those of acute form that force patients to discontinue the treatment (Miaskowski et al., 2017). Among all, the most relevant mechanism that trigger neuropathic pain (extensively reviewed by Kanat et al., 2017), is the binding of oxaliplatin to the mitochondrial DNA of sensory neurons, thus causing their death. This oxaliplatin-dependent neuronal loss also accounts for the persistence of symptoms for up to years following treatment discontinuation (Kokotis et al., 2016). The loss of sensory neurons is further confirmed by alterations in taste and smell frequently observed in patients who underwent oxaliplatin treatment (reviewed in Gamper et al., 2012). Other relevant side effects consist of fever (Saif, 2007), thrombocytopenia (Jardim et al., 2012), anemia (Cobo et al., 2007), nausea (Fleishman et al., 2012), liver function abnormalities (Lu et al., 2019), and gastro-intestinal dysfunction (Boussios et al., 2012).

This latter side effects, such as diarrhea or constipation, might be ascribed to enteric neuronal loss. In this regard, McQuade and colleagues demonstrated that the use of antioxidant molecules, such as the novel BPF-15, ameliorated the oxidative stress-dependent gastrointestinal symptoms (McQuade et al., 2016, 2018). Also, oxaliplatin can induce cognitive impairment. This debilitating condition was observed in a rat model (Fardell et al., 2012) and in patients who experienced an oxaliplatin-dependent impairment of verbal memory (Cruzado et al., 2014).

Another side effect of oxaliplatin treatment was observed by Okamoto in a Japanese patient who reported the Lhermitte’s sign, a sudden sensation resembling an electric shock that passes down the back of the neck and into the spine and may then radiate out into the arms and legs. This symptom mainly occurs in multiple sclerosis and is believed to be due to a demyelination of the posterior columns of the spinal cord induced by chemotherapy (Okamoto et al., 2020).

Finally, in a very recent study a high increase of serum neurofilament light chain level was observed in patients who referred an oxaliplatin-dependent neuropathic pain. The serological increase of neurofilament light chain has been previously reported in patient with Alzheimer’s disease, frontotemporal dementia, and multiple sclerosis. However, even if the authors concluded that a major part of the serum neurofilament light chain originates from the peripheral nervous system, it is not excluded that something has happened in the CNS, but more insights are needed (Kim et al., 2020).

## Oxaliplatin-Induced Neuropathy

Many authors have underlined the main effects exerted by oxaliplatin (Table 1) and have evidenced that one of the main causes of the neuropathy onset is its accumulation inside the cells. Within the nervous system, oxaliplatin preferentially accumulates in those cells that express specific membrane transporters such as the multidrug and toxin extrusion proteins (MATEs), the organic anion-transporting polypeptides (OATPs), and the organic cation transporter (OCT) (Huang et al., 2020). The expression of OCT-type transporters by sensory neurons of the dorsal root ganglia (DRG) (Fujita et al., 2019) not only demonstrates the accumulation of the chemotherapy mainly in these cell type, but also that this event could trigger a peripheral neurotoxicity. These data were corroborated by other results demonstrating the oxaliplatin-dependent detrimental effect on DRG neurons causing a reduced volume of the neuronal soma and an increase in the number of multinucleated neuronal nuclei (Di Cesare Mannelli et al., 2017).

**TABLE 1:** Main signaling pathways involved in oxaliplatin-dependent alterations.

	**Effects**	**Literature**
ABCs (ATP-binding cassettes)	Single nucleotide polymorphisms (e.g., rs717620, rs8187710, rs2231142 and rs1045642) inhibit the oxaliplatin efflux increasing its accumulation	Nichetti et al., 2019
OCTs (organic cation transporters)	Epigenetic modification increases the oxaliplatin influx in resistant cells	Liu et al., 2016
ROS (oxidative stress)	Overproduction/activation following mitochondrial impairment	Di Cesare Mannelli et al., 2012, 2013b, 2016; Massicot et al., 2013; De Monaco et al., 2014; Stankovic et al., 2020
ATF3 (activating transcription factor 3)	Significant increase in the DRG neurons in oxaliplatin-treated rats	Di Cesare Mannelli et al., 2013a
cAMP	Increased production after oxaliplatin-treated SH-SY5Y	Morucci et al., 2015
GAP-43 (growth associated protein 43)	Expression level down-regulation after oxaliplatin-treated SH-SY5Y	Morucci et al., 2015
CD86	Increased levels of protein expression after oxaliplatin-treated microglial cells	Branca et al., 2015
CX3CL1	Histone H4 acetylation in the *CX3CL1* promoter region induces the up-regulation of the cytokine	Huang et al., 2016
GFAP (glial fibrillary acidic protein)	Cell number increases both in spinal cord (dorsal horn) and brain cortex	Di Cesare Mannelli et al., 2013a, 2015
Iba1 (ionized calcium-binding adapter molecule 1)	Cell number increases both in spinal cord (dorsal horn) and brain cortex	Di Cesare Mannelli et al., 2013a, 2015
Resting → Activating state	Morphological shift from ramified/resting to activating microglia	Branca et al., 2015

The OCT transporter is also expressed on the luminal surface of the blood-brain barrier (BBB) micro-vessel endothelial cells, the OCT role in transporting oxaliplatin inside the cells has been demonstrated (Lin et al., 2010). In this regard, recently it has been reported that in a rat brain endothelial cell line expressing OCT protein (Friedrich et al., 2003), oxaliplatin elicits a dislocation of the Zonula Occludens-1 (ZO-1), one of the tight junction proteins that contribute to constitute the BBB (Branca et al., 2018). The increased permeability of the BBB allows the chemotherapy agent to enter the brain parenchyma, affecting both the neuronal (Park et al., 2009) and glial compartment (Lee and Kim, 2020).

As for the effects of oxaliplatin on neurons, an increase in oxidative stress is the most remarkable event. *In vitro* experiments have demonstrated that oxaliplatin is able to increase the reactive oxygen species (ROS) and the superoxide anion levels as well as protein carbonylation (Di Cesare Mannelli et al., 2013b), thus suggesting that oxidative stress could be responsible for oxaliplatin-induced neuropathic pain.

*In vivo* analysis carried out on plasma, sciatic nerves, and lumbar portion of the spinal cord obtained from oxaliplatin-treated rats strengthened the role of oxidative stress in the onset of neuropathic pain (Di Cesare Mannelli et al., 2012).

Analysis of the signaling pathway that triggers neuropathy highlighted the involvement of activating transcription factor 3 (ATF3) protein. This protein is a member of the cAMP-responsive element binding protein family (Li et al., 2019) that, following the activation of the Toll Like receptors (TLRs), regulates a signaling cascade involved in the onset of neuropathy. Also, its expression has been found significatively increased in oxaliplatin-treated nerves and DRGs (Di Cesare Mannelli et al., 2013a) that should be achieved by cAMP-increased levels, as reported by *in vitro* analysis on oxaliplatin-treated neuronal cells (Morucci et al., 2015). Moreover, an oxaliplatin-dependent alteration of the cell viability and of the expression of the growth associated protein 43 (GAP-43), a well-known marker of axon development (Morucci et al., 2015), was also reported.

With respect to the glial compartment, neuropathic pain has been linked to changes in the gene expression and secretory profile of microglia that elicits a signaling cascade resulting in neuroinflammation (Ji et al., 2016).

As demonstrated by *in vitro* analysis both in human and murine cells, oxaliplatin induced an increase in pro-inflammatory marker expression such as clusters of differentiation 86 (CD86), and morphological changes shifting from resting to activating shape (Branca et al., 2015). These effects are in accordance with *ex vivo* experiments carried out in oxaliplatin-treated rats, showing an increase in the number of cells expressing the microglial marker Iba-1 (ionized calcium-binding adapter molecule 1) in the spinal cord as well as in the basal ganglia and “pain matrix” brain areas (Di Cesare Mannelli et al., 2013a).

The onset of microglia-dependent neuroinflammation also induces the recruitment of astrocytes. Indeed, it has been demonstrated that oxaliplatin treatment causes an increase in the expression levels of glial fibrillary acidic protein (GFAP) and a modification of astrocytic shape, both in the spinal cord and in some brain areas (Di Cesare Mannelli et al., 2015).

Microglia and astrocyte activation is strictly related to pain sensitivity since the selective inhibition of one or the other cellular type prevented pain development (Di Cesare Mannelli et al., 2014). On the other hand, an indiscriminate glial cell silencing impaired the neurorestorative mechanisms promoted by these cell types (Di Cesare Mannelli et al., 2014).

Another important target of oxaliplatin-dependent toxicity is the mitochondrion whose dysfunction leads to the generation of reactive oxygen species (ROS). Although the research on the mechanisms underlying mitochondrial toxicity and ROS generation are only beginning to be analyzed, some studies have fully demonstrated that the antioxidant properties of different molecules are able to mitigate the oxaliplatin-dependent neuropathy (Di Cesare Mannelli et al., 2016). These results suggest that ROS generation and mitochondrial impairment are early events in the oxaliplatin-triggered signaling pathway that results in the onset of neuropathy.

## Genetic and Epigenetic Role in Oxaliplatin-Induced Neuropathic Pain

Over the last 10 years research has highlighted a very important role of epigenetics in determining variations that induce lasting or permanent changes in neuronal function (Borrelli et al., 2008). Also, it is now evident that drug exposure leads to epigenomic changes that are the basis of the different individual responses to chemotherapy. Indeed, the focus is now centered on improving the chemotherapeutic efficacy of anticancer molecules through pharmacogenetic and pharmacoepigenetic approaches (Mohelnikova-Duchonova, 2014).

Pharmacogenetics, recently changed to the term pharmacogenomics, is the field of research that encompasses all genes in the genome that may determine drug response (Pirmohamed, 2001). Indeed, especially for what may concern drug resistance and chemotherapy, the study of genetic polymorphisms is essential to choose the optimal personalized therapeutic treatment, minimizing the side effects produced by chemotherapy (Lesko, 2007). On the other hand, epigenetic modifications can influence the drug response and a decisive role in personalized medicine is assigned to pharmacoepigenetics (Majchrzak-Celińska, 2017).

In view of these fascinating scenarios, it has been recently hypothesized that the pivotal role of single nucleotide polymorphisms (SNPs) affects the gene coding for oxaliplatin transporters. The alteration of the expression levels and the functioning of these transporters, in particular, the SNPs occurring in ATP-binding cassette (ABCs) transporters (such as rs717620, rs8187710, rs2231142, and rs1045642), causes an increase in the oxaliplatin concentration inside the cells (in particular, the DRG neurons) that may account for an higher risk to develop an oxaliplatin-dependent neuropathy as previously reported (Nichetti et al., 2019).

It has been reported that a prolonged and high oxaliplatin intracellular accumulation, induced ROS overproduction mediated by mitochondrial impairment (Massicot et al., 2013). Thus, even if specific transporter polymorphisms are not beneficial for patients that unfortunately do not correctly excrete oxaliplatin leading to its accumulation, the simultaneous use of antioxidant molecules during a chemotherapy regimen could help to retrieve and ameliorate the oxaliplatin-induced neuropathic pain (De Monaco et al., 2014; Stankovic et al., 2020).

However, there are many other elements that influence chemotherapeutic drug sensitivity, such as the glutathione S transferase P1 (GSTP1), involved in the inactivation of platinum-DNA adducts (Kweekel et al., 2005). For example, the 105Val allele variant at exon 5 of the GSTP1 gene confers a significantly decreased risk of developing severe oxaliplatin-related neuropathy (Lecomte et al., 2006; Chen et al., 2010, p. 201; McLeod et al., 2010; Hong et al., 2011).

Moreover, genetic polymorphisms play a key role also in adjuvant therapies where opioid drugs are used in order to ameliorate oxaliplatin-induced neuropathic pain (Wang, 2014).

Despite a clear correlation between oxaliplatin-based neuropathy and individual genetic polymorphisms, pharmacotherapy based on genetic profile is not yet routinely introduced, perhaps because different polymorphisms can correlate and a wide range of genomic analysis in a larger population is needed (Peng et al., 2013; Ruzzo et al., 2015).

In the attempt to find the molecular basis for neuropathy induction and maintenance, attention has recently been turned to epigenetic mechanisms. Epigenetic-dependent alterations of gene expression are independent of DNA sequence alterations, but they are heritable and reversible. Recently, environmental stimuli have been observed to induce long-term epigenetic modifications of the gene expression profile that characterizes neuropathic pain (extensively reviewed by Penas and Navarro, 2018).

If SNPs represent a risk of developing neuropathy, epigenetic regulation of the ABC transporters expression levels may lead to a decreased risk. Indeed, these transporters regulate the oxaliplatin efflux from cells (Sparreboom et al., 2003), thus reducing its accumulation (Huo et al., 2010).

A similar fascinating result in this field was obtained by the epigenetic modification of the OCT2 transporter both *in vitro* and in xenografts. Some authors have promoted the epigenetic expression of this oxaliplatin transporter in renal cancer cells in order to increase the oxaliplatin sensitization of these cells (Liu et al., 2016). It could be argued that the OCT2 epigenetic modification could induce an oxaliplatin accumulation also into other cell compartments, including the brain, thus leading to the induction of neuropathy.

Epigenetic modifications of glial cells have also been shown to play a role in neuropathic pain. Astrocytic DNA methylation and histone modifications, two of the major epigenetic modifications, induce the production of pro-inflammatory cytokines triggering a microglia neuroinflammatory activation that, in turn, contributes to the development of neuropathy (McMahon et al., 2005; Descalzi et al., 2015; Machelska and Celik, 2016).

It has also been demonstrated that oxaliplatin treatment significantly increased the histone H4 acetylation in the *CX3CL1* promoter region in spinal cord neurons (Huang et al., 2016), inducing the up-regulation of this cytokine. *In vivo* studies have demonstrated involvement in the induction of central sensitization and acute pain behavior after oxaliplatin administration (Huang et al., 2016; Zhang et al., 2018). More recently, it has been evidenced that oxaliplatin treatment is able to increase the expression of 10–11 translocation methylcytosine dioxygenase 1 (TET1), a well-known enzyme involved in DNA demethylation. The researchers found that TET1 up-regulation indirectly acts on Homeobox A6 protein (*HOX-A6*) expression in neurons, thus becoming a pivotal target in ameliorating oxaliplatin-induced neuropathy (Deng et al., 2020).

## Concluding Remarks

The oxaliplatin-induced neuropathic pain is a deleterious side effect for patient healthcare that could lead to therapy interruption. In recent years many efforts have been made in order to both increase the oxaliplatin anti-cancer effects and ameliorate neuropathy. Hopefully, genetic and epigenetic information can help physicians toward a personalized therapeutic strategy. However, many other analyses of pharmacogenetics and epigenetics should be performed in order to corroborate and obtain useful data to seriously improve the benefit from chemotherapeutic treatment.

## Author Contributions

JB, LD, and AP conceived the structure of the manuscript and drafted the manuscript. DC, MG, and CG critically revised the manuscript. All authors contributed to the article and approved the submitted version.

## Conflict of Interest

The authors declare that the research was conducted in the absence of any commercial or financial relationships that could be construed as a potential conflict of interest.
